# Hierarchical multi-class Alzheimer’s disease diagnostic framework using imaging and clinical features

**DOI:** 10.3389/fnagi.2022.935055

**Published:** 2022-08-10

**Authors:** Yao Qin, Jing Cui, Xiaoyan Ge, Yuling Tian, Hongjuan Han, Zhao Fan, Long Liu, Yanhong Luo, Hongmei Yu

**Affiliations:** ^1^Department of Health Statistics, School of Public Health, Shanxi Medical University, Taiyuan, China; ^2^Department of Neurology, First Hospital of Shanxi Medical University, Taiyuan, China; ^3^Center of Translational Medicine, School of Basic Medical Sciences, Shanxi Medical University, Taiyuan, China; ^4^Shanxi Provincial Key Laboratory of Major Diseases Risk Assessment, Taiyuan, China

**Keywords:** Alzheimer’s disease, diagnosis, multi-class classification, magnetic resonance imaging, surface-based morphometry

## Abstract

Due to the clinical continuum of Alzheimer’s disease (AD), the accuracy of early diagnostic remains unsatisfactory and warrants further research. The objectives of this study were: (1) to develop an effective hierarchical multi-class framework for clinical populations, namely, normal cognition (NC), early mild cognitive impairment (EMCI), late mild cognitive impairment (LMCI), and AD, and (2) to explore the geometric properties of cognition-related anatomical structures in the cerebral cortex. A total of 1,670 participants were enrolled in the Alzheimer’s Disease Neuroimaging Initiative (ADNI) database, comprising 985 participants (314 NC, 208 EMCI, 258 LMCI, and 205 AD) in the model development set and 685 participants (417 NC, 110 EMCI, 83 LMCI, and 75 AD) after 2017 in the temporal validation set. Four cortical geometric properties for 148 anatomical structures were extracted, namely, cortical thickness (CTh), fractal dimension (FD), gyrification index (GI), and sulcus depth (SD). By integrating these imaging features with Mini-Mental State Examination (MMSE) scores at four-time points after the initial visit, we identified an optimal subset of 40 imaging features using the temporally constrained group sparse learning method. The combination of selected imaging features and clinical variables improved the multi-class performance using the AdaBoost algorithm, with overall accuracy rates of 0.877 in the temporal validation set. Clinical Dementia Rating (CDR) was the primary clinical variable associated with AD-related populations. The most discriminative imaging features included the bilateral CTh of the dorsal part of the posterior cingulate gyrus, parahippocampal gyrus (PHG), parahippocampal part of the medial occipito-temporal gyrus, and angular gyrus, the GI of the left inferior segment of the insula circular sulcus, and the CTh and SD of the left superior temporal sulcus (STS). Our hierarchical multi-class framework underscores the utility of combining cognitive variables with imaging features and the reliability of surface-based morphometry, facilitating more accurate early diagnosis of AD in clinical practice.

## Introduction

The total number of people experiencing dementia worldwide is estimated to increase from 57.4 million in 2019 to 153 million in 2050 (GBD 2019 Dementia Forecasting Collaborators, 2022). Alzheimer’s disease (AD) is a major cause of disability and dependency among the elderly. Currently, there is a lack of effective treatment to slow AD progression, and autopsy constitutes the only medically confirmed diagnosis of AD, highlighting the urgent need for early diagnosis (Alzheimer’s Association, 2022).

As an established precursor of AD, mild cognitive impairment (MCI) can be divided into early mild cognitive impairment (EMCI) and late mild cognitive impairment (LMCI), according to the degree of episodic memory impairment (Aisen et al., 2010). Individuals with LMCI present with more severe cognitive impairment compared to those with EMCI (Aisen et al., 2015). However, various resources exist for pooling patients with either EMCI or LMCI into a single large MCI group, thereby precluding a better understanding of the underlying mechanisms for MCI progression (Moore et al., 2019). Despite significant efforts to ensure a rapid and rigorous diagnosis of AD, personalized multi-class diagnosis across the entire spectrum of AD remains a significant challenge. The accuracy of early diagnosis of AD remains unsatisfactory and warrants further research, due to the nature of the clinical continuum (Aisen et al., 2010).

The deep folds of the cerebral cortex allow half to two-thirds of the cortical surface to be hidden in the sulci and lateral fossa (Essen, 2005). Even trained anatomists may find it challenging to manually label sulcogyral structures in the complex folded anatomy of the cerebral cortex. Alzheimer’s disease is a progressive disease that typically invades spatially adjacent rather than isolated areas (Vemuri et al., 2008). Therefore, given the vulnerability of cortical regions to AD-related pathological changes, careful consideration of local spatial continuity and precise localization of sulcogyral structures in the cerebral cortex may be more conducive to interpret morphological and functional changes during AD progression (Liu et al., 2015). At present, the relationship between cortex geometry and cognitive dysfunction remains obscure.

We hypothesized that machine learning (ML) approaches applied to subsets of neuroimaging and clinical variables could distinguish between AD-related populations. The objectives of this study were: (1) to develop an effective classification framework for clinical populations, namely, normal cognition (NC), EMCI, LMCI, and AD and (2) to explore the geometric properties of cognition-related anatomical structures in the cerebral cortex.

## Materials and methods

### Study sample

This study used data from the Alzheimer’s Disease Neuroimaging Initiative (ADNI) database^1^. The ADNI was launched in 2003 as a public–private partnership, led by Principal Investigator Michael W. Weiner, MD. The primary goal of ADNI has been to test whether serial magnetic resonance imaging (MRI), positron emission tomography (PET), other biological markers, and clinical and neuropsychological assessment can be combined to measure the progression of MCI and early AD. For the ADNI study, written informed consent was obtained for all participants and the study protocol was approved by the institutional review board at each participating center before protocol-specific procedures were performed.

Taking 2017 as the cut-off time point, the data from the ADNI database were divided into two parts: the “model development set” and the “temporal validation set.” For the model development set, we screened participants on the basis of structural MRI scans and corresponding MMSE scores at four-time points after their initial visit; the cognitive state of all participants remained stable over time, including those with NC EMCI, LMCI, and AD. A total of 1,670 participants were enrolled in this study, comprising 985 participants (314 NC, 208 EMCI, 258 LMCI, and 205 AD) in the model development set and 685 participants (417 NC, 110 EMCI, 83 LMCI, and 75 AD), enrolled after 2017, in the temporal validation set. Demographic characteristics (age, sex, length of education, and marital status), apolipoprotein E (APOE) genotypes, and clinical assessment scores [Clinical Dementia Rating (CDR) and Functional Activities Questionnaire (FAQ)] at baseline were obtained for all participants (Table 1).

**TABLE 1:** Demographic and clinical assessments in the model development and temporal validation sets.

Data set	Variable	NC (*n* = 314)	EMCI (*n* = 208)	LMCI (*n* = 258)	AD (*n* = 205)
Model development (*n* = 985)	Age (years)	74.22 ± 5.73	71.34 ± 7.58	73.49 ± 7.36	74.62 ± 7.88
	Sex (male)	160 (50.96)	88 (42.31)	105 (40.70)	94 (45.85)
	Length of education (years)	16.23 ± 2.69	16.11 ± 2.75	15.98 ± 2.82	15.24 ± 2.88
	Marital status (married)	214 (68.15)	161 (77.40)	194 (75.19)	172 (83.90)
	APOEε4 carriers	74 (23.57)	66 (31.73)	90 (34.88)	98 (47.80)
	CDR	0.04 ± 0.14	1.21 ± 0.69	1.45 ± 0.85	4.26 ± 1.53
	FAQ	0.17 ± 0.69	1.88 ± 3.02	2.76 ± 3.75	12.92 ± 6.51
	MMSE	29.19 ± 1.04	28.28 ± 1.57	27.68 ± 1.69	23.24 ± 2.30

**Temporal validation (*n* = 685)**	**Variable**	**NC (*n* = 417)**	**EMCI (*n* = 110)**	**LMCI (*n* = 83**)	**AD (*n* = 75)**

	Age (years)	70.94 ± 6.22	71.16 ± 6.66	71.70 ± 8.20	73.90 ± 8.00
	Sex (male)	242 (58.03)	47 (42.73)	34 (40.96)	31 (41.33)
	Length of education (years)	16.85 ± 2.33	16.18 ± 2.76	16.08 ± 2.66	15.75 ± 2.48
	Marital status (married)	307 (73.62)	88 (80.00)	61 (73.49)	59 (78.67)
	APOEε4 carriers	132 (31.65)	37 (33.64)	35 (42.17)	45 (60.00)
	CDR	0.07 ± 0.25	1.22 ± 1.02	1.54 ± 1.02	5.61 ± 2.79
	FAQ	0.24 ± 0.89	2.41 ± 4.09	3.46 ± 3.84	15.55 ± 8.04
	MMSE	29.10 ± 1.15	28.26 ± 1.77	27.48 ± 2.18	21.87 ± 4.49

NC, normal cognition; EMCI, early mild cognitive impairment; LMCI, late mild cognitive impairment; AD, Alzheimer’s disease; CDR, clinical dementia rating; FAQ, functional activities questionnaire; MMSE, mini-mental state exam.

The general inclusion/exclusion criteria were as follows: participants in the NC group had a Mini-Mental State Examination (MMSE) score between 24 and 30 (inclusive) and a CDR score of 0, without significant impairments in cognition or activities of daily living. Early mild cognitive impairment participants exhibited mild cognitive decline, with a CDR score of 0.5, MMSE score between 24 and 30 (inclusive), and objective memory loss as identified using the delayed recall of one paragraph from the Wechsler Memory Scale Logical Revised Memory II (WMS-R II) (adjusted for age and length of education: ≥16 years, 9–11; 8–15 years, 5–9; 0–7 years, 3–6). Late mild cognitive impairment participants had poorer objective memory, as measured with the WMS-R II (adjusted for age and length of education: ≥16 years, ≤8; 8–15 years, ≤4; 0–7 years, ≤2). The AD diagnosis was based on the NINCDS/ADRDA criteria. For more detailed information, refer to http://www.adni-info.org/Scientists/ADNIGrant/ProtocolSummary.aspx.

### Overview of the multi-class framework

**FIGURE 1:**
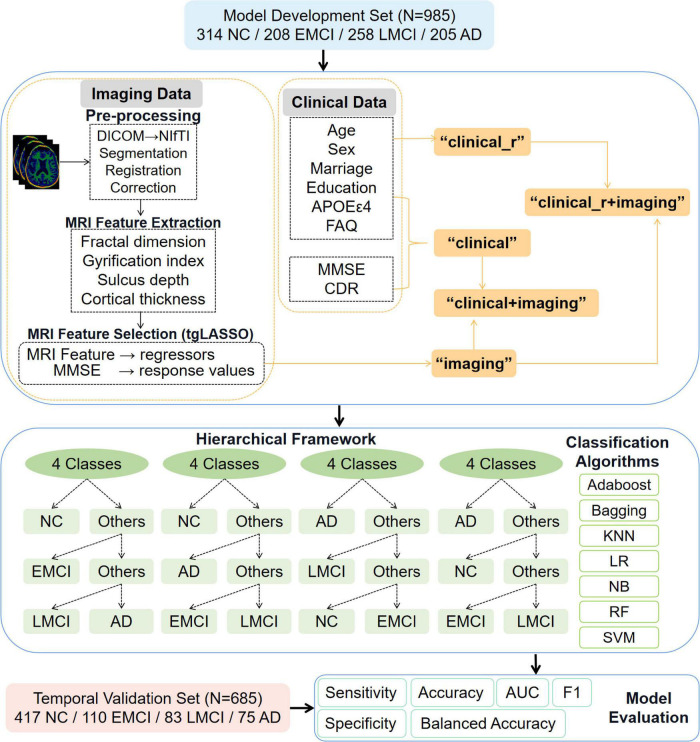
Overview of the hierarchical multi-class framework. AD, Alzheimer’s disease; MCI, mild cognitive impairment; EMCI, early mild cognitive impairment; LMCI, late mild cognitive impairment; NC, normal cognition; MRI, magnetic resonance imaging; DICOM, digital imaging and communications in medicine; NIFTI, neuroimaging informatics technology initiative; CDR, Clinical Dementia Rating; FAQ, Functional Activities Questionnaire; MMSE, mini-mental state exam; KNN, K-nearest neighbor; LR, logistic regression; NB, naive Bayes; RF, random forest; SVM, support vector machine; AUC, area under the curve.

The multi-class framework consisted of three parts: MRI feature extraction, optimal feature subset selection, and hierarchical multi-class classification, as shown in Figure 1. First, a fully conditional specification method was used for multiple imputations of missing data of clinical features, and we extracted the cortical geometric properties of each anatomical structure from neuroimaging scans in the entire data set. Second, imaging data in the model development set were integrated with MMSE scores at the corresponding time points to capture discriminative imaging features by introducing a regression task. Third, based on the selected imaging features, clinical variables, and their multiple combinations at baseline, several ML algorithms and 10-fold cross-validation were used to implement a hierarchical four-way classification for the model development set, and the optimal model was applied to the temporal validation set for blind testing.

### Magnetic resonance imaging acquisition

All structural MRI scans were converted from raw Digital Imaging and Communications in Medicine files to the Neuroimaging Informatics Technology Initiative format using MRIcro software. Subsequently, all images were preprocessed and subjected to motion correction, non-brain tissue removal, segmentation, intensity normalization, tessellation of gray and white matter boundaries, topology correction, and spatial smoothing using CAT12^2^ operated in SPM12^3^ and implemented in MATLAB 2013a. Central surface evaluation algorithms can automatically correct artifacts and defects during reconstruction, and the results were not different from those obtained using FreeSurfer, supporting the credibility of our findings (Yotter et al., 2011a; Dahnke et al., 2013).

### Magnetic resonance imaging feature extraction

We used the Destrieux parcellation protocol proposed in August 2009 (Destrieux et al., 2010), which involves complete parcellation of cortical surfaces with anatomical rules and nomenclature available in the FreeSurfer package (FreeSurfer v4.5, aparc.a2009s), with 74 anatomical structures per hemisphere. We calculated four cortical geometric properties corresponding to each anatomical structure, namely, cortical thickness (CTh), fractal dimension (FD), gyrification index (GI), and sulcus depth (SD). The CTh calculation adopted an automatic projection-based thickness measurement method (Dahnke et al., 2013). Sulcus depth was calculated according to the Euclidean distance between the central surface and its convex hull. The GI and FD were calculated based on absolute mean curvature and spherical harmonics, respectively (Yotter et al., 2011b). In total, 592 imaging features were obtained for each participant at each time point.

### Magnetic resonance imaging feature selection

Given the high dimensionality and poor accessibility of longitudinal neuroimaging data, sparse regression methods are widely used for feature dimension reduction (Yang et al., 2019). In the current study, imaging features and MMSE scores in the model development set were regarded as regressors and target response values, respectively. We used temporally constrained group sparse learning (tgLASSO) to create regression models with the aim of selecting the optimal subset of imaging features for subsequent classification tasks (Zhang et al., 2012). Each subject has different imaging features at *T* time points. *X*_*j*_ and *y*_*j*_ denote the imaging features and corresponding MMSE scores, respectively. Here, the key goal of tgLASSO was to incorporate the group regularization and smoothness regularization terms into the objective function: $\min_{W}=J\,\left(W\right)=\frac{1}{2}\,\sum_{j=1}^{T}{||y_{j}-X_{j}\,w_{j}||}_{2}^{2}+R_{g}\,\left(W\right)+R_{s}\,\left(W\right)$. The group regularization parameter *R*_*g*_(*W*) = λ_1_||*w*||_2,1_ controlled the group sparsity of the linear models. Imaging features from multiple time points were employed to combine the weights of different time points in the same anatomical region with the regularization item, to jointly select features based on the strength of different time points. Further, two smooth regularization terms were added to the objective function to reflect smooth changes between data from adjacent time points: Rs(W)=λ2∑j=1T-1||wj-wj+1||1+λ∑j=1T-13||Xjwj-Xj+1$w_{j+1}|{|}_{2}^{2}$. The fused smoothness term $\lambda_{2}\sum_{j=1}^{T-1}||w_{j}-w_{j+1}||{}_{1}$, which originated from fused LASSO, constrained small differences between two successive weight vectors from adjacent time points (Zille et al., 2017). The output smoothness termλ3∑|j=1T-1|Xjwj-Xj+1wj+1||22, which also required small differences between the outputs of two successive models from adjacent time points (i.e., the anatomical structures sensitive to different stages of AD), was filtered out. These two smoothness regularization terms balanced the relative contributions and controlled the smoothness of the linear models. It should be noted that the tgLASSO method was only used for MRI feature selection in the model development set and not for the entire data set. After a number of attempts, the final regularization parameters λ_1_, λ_2_, and λ_3_ were set at 0.25, 0.08, and 0.04, respectively.

### Multi-class classification

#### Hierarchical framework

The optimal subset of cognition-related imaging features was selected–using the tgLASSO method–as the “imaging” features for the classification tasks. Demographic characteristics, APOE genotypes, and clinical assessment scores (FAQ, MMSE, and CDR) were combined as “clinical” features. The combination of the above two feature types then yielded new features, which we labeled “clinical + imaging” features. Considering that CDR and MMSE scores were key characteristics used to categorize participants in the ADNI database, we added two classification features for our sensitivity analysis. The “clinical_r” features referred to the “clinical” features except MMSE and CDR scores, and the “clinical_r + imaging” features referred to the combination of “clinical_r” and “imaging” features. We created four hierarchical multi-class scenarios and transformed the four-way classification into three binary classification tasks using a hierarchical process, as shown in Figure 1.

The four hierarchical multi-class scenarios were “NC-EMCI-LMCI-AD,” “AD-LMCI-NC-EMCI,” “AD-LMCI-NC-EMCI,” and “AD-NC-EMCI-LMCI.” For example, in the AD-LMCI-NC-EMCI scenario, AD was considered one class, and NC, EMCI, and LMCI were considered another class (“Others”). These two classes were trained on the first classifier to obtain AD candidates. Subsequently, LMCI was considered one class, and NC and EMCI were considered another class. These two classes were trained on the second classifier to obtain LMCI candidates. Finally, the third classifier was trained to distinguish NC from EMCI. The final classification results for each participant were obtained using these binary classifiers.

Given that the sample imbalance in multiple binary classifications tends to result in suboptimal classification performance, the synthetic minority oversampling technique was embedded to resample raw features in the model development set and to create synthetic minority class samples for improving model performance (Chawla et al., 2011). The minority class was oversampled by introducing random linear interpolation between each data sample point and its k-nearest neighbors (KNNs). In this study, k was set at 10. We implemented different classification tasks based on the five features (i.e., “clinical,” “clinical_r,” “imaging,” “clinical + imaging,” and “clinical_r + imaging”) defined earlier in four different scenarios, and evaluated and compared classification performance.

#### Classification algorithms

Machine learning can overcome the “dimensionality curse” and thus permits the learning of complex and subtle changes from well-generalized training samples, thereby enabling us to identify patterns in new test samples (Mishra and Li, 2020). We employed multiple ML methods for model development, that is, AdaBoost, bagging, k-nearest neighbor, logistic regression (LR), naive Bayes (NB), random forest (RF), and support vector machine (SVM) algorithms.

AdaBoost is an ensemble learning algorithm based on boosting, characterized by sequential training of base classifiers (Vong and Du, 2020). At each iteration, the weight distribution of training samples is considered to ensure that larger weights are featured to misclassified samples under the earlier iterations, and final classification results are obtained by weighted majority voting of base classifiers.

Bagging classifiers use the bootstrap method to create various data subsets from the main training data, and final outputs are voted by all base classifiers learning in parallel (Lin et al., 2022).

The KNN method is an extension of the nearest neighbor algorithm based on supervised learning, which compares test samples with similar training samples through analogical learning, and describes “closeness” using distance metrics like Euclidean distances (Hu et al., 2016). Classification results are determined by a majority vote of k neighbors.

An LR algorithm is a statistical probabilistic binary classifier that applies the logit function to perform linear transformations to obtain the highest posterior probability of one of the two classes.

Naive Bayes classifiers are probabilistic classifiers based on Bayes’ theorem, which estimates the prior probability of training samples belonging to each class and the posterior probability of test samples belonging to each class, and then classifies them according to the maximum posterior probability (Sugahara and Ueno, 2021).

Random forest algorithms represent an ensemble of different decision trees, whose main parameters are the number of trees in the “forest” and variables used in the node decision split. Each node split usually depends on different subsamples of randomly selected features (Rigatti, 2017).

Support vector machine projects the target data into a high-dimensional space through kernel functions to generate the optimal hyperplane, which maximizes the marginal distance for both classes and minimizes the classification error. The support vectors are the data points in each class that come closest to the hyperplane and form the margin boundary.

For each algorithm of the four hierarchical multi-class scenarios in the model development set, we tested a series of values for the tuning procedures and determined the optimal parameters based on the model performance. The training and test sets in the model development set were adequately separated using 10-fold cross-validation, where the training set in each cross-validation iteration was resampled, whereas the test set was only used to test the classification performance and obtain the optimal model.

### Model evaluation and temporal validation

Seven metrics were quantified to compare the performance of imaging features, clinical variables, and their multiple combinations: sensitivity, specificity, accuracy, balanced accuracy, F1 score, and area under the curve (AUC). The temporal validation set was devoted to a final blindfolded evaluation of the optimal model from the model development set. The overall accuracy was the proportion of the four AD-related populations correctly classified in the time verification set.

Sensitivity=TP⁢/⁢(TP+FN)

Specificity=TN⁢/⁢(TN+FP)

Accuracy=(TP+TN)⁢/⁢(TP+FP+TN+FN)

Balanced⁢accuracy=(Sensitivity+Specificity)⁢/⁢2

F1⁢score= 2×TP⁢/⁢(2×TP+FP+FN)

where TP, true positive; TN, true negative; FP, false positive; FN, false negative.

## Results

### Discriminative features

The degree of contribution of 40 discriminative features was obtained by the dimension reduction of imaging features. The specific weight values presented in Table 2 show that the geometric properties of the top 10 different anatomical structures are the FD of the lS_occipital_ant, the GI of the rG_octemp_medParahip, and rG_cingulPostventral, followed by the GI of the S_octemp_med_and_Lingual, the FD of the rS_circular_insula_inf, lG_temp_supLateral, rS_oc_sup_and_transversal, rG_cingul-Post-dorsal, and the CTh of the lS_orbitalH_Shaped and rG_pariet_infAngular.

**TABLE 2:** Weight values of forty most important features by the dimension reduction of imaging features using the Destrieux parcellation protocol proposed in August 2009 (FreeSurfer v4.5, aparc.a2009s).

aparc.a2009s Index	Short name	Anatomical name	Hemisphere	Geometric properties	*w*	|ln|*w*||	Weight rank
6	G_and_S_cingulAnt	Anterior part of cingulate gyrus and sulcus	Right	SD	−4.038*e*−3	5.512	40
7	G_and_S_cingulMidAnt	Middle-anterior part of cingulate gyrus and sulcus	Right	SD	−2.651*e*−3	5.933	33
9	G_cingul-Post-dorsal	Posterior-dorsal part of cingulate gyrus	Left	CTh	2.12*e*−3	6.158	32
				FD	2.95*e*−4	8.128	8
			Right	CTh	6.672*e*−4	7.312	16
10	G_cingulPostventral	Posterior-ventral part of the cingulate gyrus (isthmus of the cingulate gyrus)	Right	GI	4.792*e*−5	9.946	3
13	G_front_infOrbital	Orbital part of inferior frontal gyrus	Left	CTh	−4.306*e*−4	7.750	11
17	G_Ins_lg_and_S_ cent_ins	Long insular gyrus and insulacentral sulcus	Left	FD	7.729*e*−4	7.165	18
23	G_octemp_medParahip	Parahippocampal gyrus, parahippocampal part of medial occipito-temporal gyrus	Left	CTh	9.782*e*−4	6.930	21
			Right	CTh	3.16*e*−3	5.758	37
				GI	−3.70*e*−5	10.204	2
25	G_pariet_infAngular	Angular gyrus	Left	CTh	1.237*e*−3	6.695	25
			Right	CTh	3.828*e*−4	7.868	10
34	G_temp_supLateral	Lateral aspect of superior temporal gyrus	Left	FD	1.362*e*−4	8.901	6
35	G_temp_supPlan_polar	Planum polare of superior temporal gyrus	Left	GI	5.301*e*−4	7.543	12
			Right	GI	6.002*e*−4	7.418	13
41	Lat_Fispost	Posterior ramus (or segment) of lateral sulcus (or fissure)	Left	SD	1.681*e*−3	6.388	29
			Right	GI	7.468*e*−4	7.200	17
42	Pole_occipital	Occipital pole	Right	SD	6.576*e*−4	7.327	15
43	Pole_temporal	Temporal pole	Right	GI	−8.033*e*−4	7.127	19
46	S_cingulMarginalis	Marginal branch (or part) of cingulate sulcus	Left	FD	3.597*e*−3	5.628	38
48	S_circular_insula_inf	Inferior segment of circular sulcus of insula	Left	GI	1.507*e*−3	6.497	28
			Right	FD	−1.220*e*−4	9.011	5
				GI	2.775*e*−3	5.887	35
49	S_circular_insula_sup	Superior segment of circular sulcus of insula	Left	FD	1.101*e*−3	6.812	23
				GI	1.407*e*−3	6.566	26
			Right	FD	8.454*e*−4	7.076	20
				GI	2.718*e*−3	5.908	34
58	S_oc_sup_and _transversal	Superior occipital sulcus and transverse occipital sulcus	Right	FD	2.724*e*−4	8.208	7
59	S_occipital_ant	Anterior occipital sulcus and preoccipital notch (temporo-occipital incisure)	Left	FD	2.906*e*−5	10.446	1
			Right	FD	1.063*e*−3	6.846	22
60	S_octemp_lat	Lateral occipito-temporal sulcus	Right	FD	1.707*e*−3	6.373	30
61	S_octemp_med_and _Lingual	Medial occipito-temporal sulcus (collateral sulcus) and lingual sulcus	Left	GI	1.191*e*−4	9.035	4
64	S_orbitalH_Shaped	Orbital sulci (H-shaped sulci)	Left	CTh	−3.028*e*−4	8.102	9
69	S_precentralsuppart	Superior part of precentral sulcus	Left	FD	6.093*e*−4	7.403	14
73	S_temporal_sup	Superior temporal sulcus (parallel sulcus)	Left	CTh	1.218*e*−3	6.711	24
				FD	1.506*e*−3	6.498	27
				SD	3.923*e*−3	5.541	39
			Right	FD	2.041*e*−3	6.194	31
				SD	3.006*e*−3	5.807	36

CTh, cortical thickness; FD, fractal dimension; GI, gyrification index; SD, sulcus depth.

### Classification performance

**FIGURE 2:**
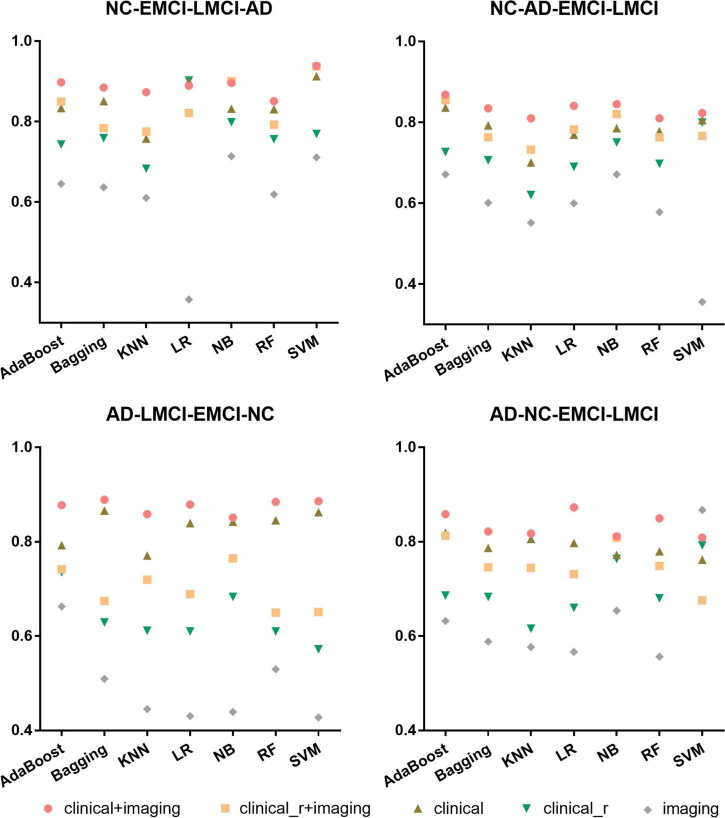
Overall accuracy of the temporal validation set in four scenarios using seven machine learning (ML) algorithms. AD, Alzheimer’s disease; EMCI, early mild cognitive impairment; LMCI, late mild cognitive impairment; NC, normal cognition; KNN, K-nearest neighbor; LR, logistic regression; NB, naive Bayes; RF, random forest; SVM, support vector machine.

**FIGURE 3:**
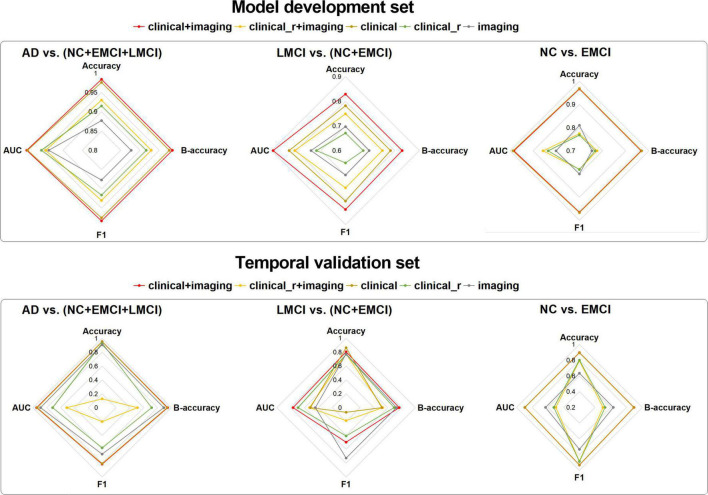
Radar charts of binary classification tasks based on imaging features, clinical variables, and their multiple combinations in the “AD-LMCI-EMCI-NC” scenario using the AdaBoost algorithm. AD, Alzheimer’s disease; EMCI, early mild cognitive impairment; LMCI, late mild cognitive impairment; NC, normal cognition; KNN, K-nearest neighbor; LR, logistic regression; NB, Naive Bayes; RF, random forest; SVM, support vector machine; B-accuracy, balanced accuracy, AUC, area under the curve.

The overall accuracy of multiple combinations of different classification features and ML algorithms in the temporal validation set is shown in Figure 2. Among the four hierarchical multi-class scenarios, the “clinical+imaging” features showed the greatest improvement in overall accuracy, all above 0.8, thereby demonstrating the superiority and necessity of the combination. The “clinical_r + imaging” and “clinical” features came next, exhibiting a difference in overall accuracy to the “clinical + imaging” features of 0.001–0.235 and 0.009–0.115, respectively. The overall accuracy for “clinical_r” features alone ranged from 0.6 to 0.8, while the “imaging” features performed poorly. Regardless of the classification scenario, AdaBoost always maintained a more robust performance than the other algorithms, with relatively small overall accuracy differences among different features. Details of all classification results using AdaBoost are provided in the Supplementary Material (Tables 1–4). For the current study, we only used the robust classification results of the AdaBoost applied to the AD-LMCI-NC-EMCI scenario as an example (see the radar charts in Figure 3). The “clinical + imaging” features still performed best in multiple binary classification tasks, followed by the “clinical” features.

For the binary classification task AD vs. (NC + EMCI + LMCI) in the model development set, all evaluation indicators were above 0.85. The performance of the “clinical + imaging” features was generally similar to that of the “clinical” features, close to one. Although the AUC of the “clinical_r + imaging” features was smaller than that of the “clinical_r” features, the former performed better on the whole. The AUC of the “imaging” features was approximately 0.94. In the temporal validation set, the performance of the “clinical + imaging” was better but still similar to that of the “clinical” features. The performance of the “imaging” features was higher than that of the “clinical_r” and “clinical_r + imaging” features. The “clinical_r + imaging” had a lower accuracy and F1 score.

For the binary classification task LMCI vs. (NC + EMCI) in the model development set, the order of the evaluation indicators for the different features was clear: “clinical + imaging” > “clinical” > “clinical_r + imaging” > “imaging” > “clinical_r.” The AUC of the “clinical + imaging” features was approximately 0.9. In the temporal validation set, the accuracy of the different kinds of features was similar. The AUC and balanced accuracy of the “clinical+imaging” features were the highest, while the “imaging” features had the highest F1 score.

For the binary classification task NC vs. EMCI, the “clinical+imaging” and clinical features had almost the same performance in both the model development and the time validation set, and the same was found for the “clinical_r + imaging” and “clinical_r” features. The accuracy and F1 scores of the “imaging” features in the model development set were higher than those of the “clinical_r + imaging” and “clinical_r” features, while the AUC and balanced accuracy were higher in the time verification set.

In sum, “clinical + imaging” feature combination improved the classification performance of the AdaBoost algorithm, with an overall accuracy of 0.877 in the temporal validation set (Table 3).

**TABLE 3:** Hierarchical multi-class results of imaging features, clinical variables, and their multiple combinations in the “AD-LMCI-NC-EMCI” scenario using the AdaBoost algorithm (clinical_r refers to clinical features removing MMSE and CDR).

Dataset	Features	Classifiers	SEN	SPE	Accuracy	B-accuracy	F1	AUC
Model development	Clinical+imaging	AD vs. (NC + EMCI + LMCI)	0.996	0.972	0.984	0.984	0.983	0.994
		LMCI vs. (NC + EMCI)	0.895	0.765	0.829	0.830	0.839	0.894
		NC vs. EMCI	0.934	1.000	0.967	0.967	0.965	0.983
	Clinical_r+imaging	AD vs. (NC + EMCI + LMCI)	0.942	0.916	0.930	0.929	0.930	0.945
		LMCI vs. (NC + EMCI)	0.757	0.742	0.749	0.750	0.751	0.806
		NC vs. EMCI	0.800	0.751	0.774	0.776	0.780	0.857
	Clinical	AD vs. (NC + EMCI + LMCI)	0.979	0.972	0.976	0.976	0.975	0.992
		LMCI vs. (NC + EMCI)	0.903	0.662	0.782	0.783	0.805	0.829
		NC vs. EMCI	0.937	1.000	0.969	0.969	0.967	0.987
	Clinical_r	AD vs. (NC + EMCI + LMCI)	0.947	0.884	0.915	0.916	0.916	0.956
		LMCI vs. (NC + EMCI)	0.620	0.723	0.671	0.672	0.650	0.718
		NC vs. EMCI	0.828	0.707	0.768	0.768	0.781	0.835
	Imaging	AD vs. (NC + EMCI + LMCI)	0.890	0.863	0.877	0.877	0.877	0.937
		LMCI vs. (NC + EMCI)	0.707	0.685	0.697	0.696	0.699	0.740
		NC vs. EMCI	0.580	0.618	0.600	0.599	0.590	0.646
Temporal validation	Clinical+imaging	AD vs. (NC + EMCI + LMCI)	0.933	0.956	0.953	0.945	0.814	0.945
		LMCI vs. (NC + EMCI)	0.711	0.820	0.805	0.766	0.498	0.765
		NC vs. EMCI	0.897	0.891	0.896	0.894	0.932	0.894
	Clinical_r+imaging	AD vs. (NC + EMCI + LMCI)	1.000	0.020	0.127	0.510	0.201	0.510
		LMCI vs. (NC + EMCI)	0.193	0.863	0.772	0.528	0.187	0.528
		NC vs. EMCI	1.000	0.000	0.791	0.500	0.883	0.500
	Clinical	AD vs. (NC + EMCI + LMCI)	0.920	0.956	0.952	0.938	0.807	0.938
		LMCI vs. (NC + EMCI)	0.036	0.994	0.864	0.515	0.067	0.515
		NC vs. EMCI	0.894	0.890	0.894	0.892	0.930	0.893
	Clinical_r	AD vs. (NC + EMCI + LMCI)	0.440	0.990	0.930	0.715	0.579	0.715
		LMCI vs. (NC + EMCI)	0.590	0.795	0.767	0.693	0.408	0.693
		NC vs. EMCI	0.998	0.055	0.801	0.527	0.888	0.526
	Imaging	AD vs. (NC + EMCI + LMCI)	0.867	0.912	0.907	0.890	0.670	0.889
		LMCI vs. (NC + EMCI)	0.663	0.791	0.774	0.727	0.727	0.444
		NC vs. EMCI	0.635	0.627	0.634	0.631	0.733	0.631

AD, Alzheimer’s disease; EMCI, early mild cognitive impairment; LMCI, late mild cognitive impairment; NC, normal cognition; SEN, sensitivity; SPE, specificity; B-accuracy, balanced accuracy; AUC, area under the curve.

### Feature importance

In the AD-LMCI-NC-EMCI scenario, the RF algorithm generated the feature importance scores via an out-of-bag error estimate among the binary classification tasks using “clinical + imaging” features, as shown in the Supplementary Material (Figure 1). The mean importance scores of the clinical features were above 20 on the three binary tasks, significantly higher than those of the imaging features. Clinical dementia rating scores were primarily associated with AD multi-class classification, with feature importance scores of up to 85 for the binary classification task NC vs. EMCI. For the binary classification task AD vs. (NC + EMCI + LMCI), the top five important imaging features were the CTh of the bilateral G_octemp_medParahip and G_pariet_infAngular and left S_temporal_sup. For the binary classification task LMCI vs. (NC + EMCI), the top five important imaging features were the CTh of the bilateral G_cingul-Post-dorsal and G_octemp_medParahip and the SD of the left S_temporal_sup. For the binary classification task NC vs. EMCI, the important imaging features were the CTh of the right G_pariet_infAngular and the left S_temporal_sup and the GI of the left S_circular_insula_inf.

In brief, each binary classifier exhibited good discriminative ability, and combined features improved the classification performance of the hierarchical multi-class framework.

## Discussion

In this study, a hierarchical multi-class framework for the auxiliary diagnosis of AD was created using combined clinical and imaging features, with an overall accuracy of 0.877 in the temporal validation set. The CDR score was the primary clinical variable associated with AD-related populations. The most discriminative imaging features included the bilateral CTh of the dorsal part of the posterior cingulate gyrus, parahippocampal gyrus (PHG), parahippocampal part of the medial occipito-temporal gyrus, and angular gyrus, the GI of the left inferior segment of the insula circular sulcus, and the CTh and SD of the left superior temporal sulcus (STS).

### Brain surface research

Cortical surface properties extracted in a vertex-wise manner can identify the neuroanatomical differences among different AD-related populations (Ma et al., 2020; Basheera and Ram, 2020) and provide important Supplementary information about the shape of brain structures rather than size (e.g., volume) (Ieva et al., 2015). Surface-based morphometry has the advantages of not only being visually simplified by inflation and fully automated labeling of MRI scans, which provides better repeatability and practicality (Yotter et al., 2011c) but also of using cortical geometry to drive cross-disciplinary registration, thereby fully accounting for individual differences in cortical anatomy (Fischl et al., 2015).

Previous studies have suggested that cortical folding is associated with cognitive function in the elderly (Liu et al., 2012). King et al. (2010) have discovered the potential of FD as a quantitative marker of cerebral cortical structure in mild AD. Núñez et al. (2020) reported that a higher GI of the insular cortex was strongly associated with better memory function and semantic fluency only in patients with AD. Further, Park et al. (2012) found that SD may contain important information for distinguishing AD from MCI. Im et al. (2008) suggested that patients with MCI and AD exhibited a significantly shallower SD compared to NC. To our best knowledge, the GI and SD are less widely investigated in AD-related studies compared to CTh, and less attention has been paid to cortical morphological measurements in classification tasks. The GI and SD included in this study can, therefore, serve as good measures of cortical folding complexity. Notably, the geometric properties of the anatomical structures identified in this study may permit more comprehensive indexing of relevant information in the cerebral cortex.

### Important feature contribution

Neuroimaging techniques may facilitate the tracking of disease progression due to their excellent spatial resolution, high availability, noninvasive nature, and ability to contrast different soft tissues (Altaf et al., 2018). Schwarz et al. (2016) have recommended a composite of thickness of the PHG, angular gyrus, and temporal lobe as a signature measurement for AD. A 2012 meta-analysis revealed extensive gray matter defects in the PHG, temporal lobe, cingulate gyrus, and insular cortex in patients with AD (Vasconcelos et al., 2011). Dickerson et al. (2001) have failed to identify significant atrophy of PHG in patients with very mild AD, while Echávarri et al. (2011) proposed that PHG is a highly sensitive discriminator for detecting AD, especially during the preclinical phase. Similarly to the latter, we observed that not only the PHG but also the bilateral CTh in the parahippocampal part of the medial occipito-temporal gyrus were extremely important imaging features in both the AD vs. (NC + EMCI + LMCI) and the LMCI vs. (NC + EMCI) classification tasks, as was the right CTh in the angular gyrus for the NC vs. EMCI classification task.

The posterior cingulate cortex is a highly connected and metabolically active brain region, appearing as a particularly sensitive hub for the pathological progression of AD. Lehmann et al. (2010) detected a decrease in CTh in the posterior cingulate cortex in AD pathology, and Mutlu et al. (2016) observed hypometabolism and atrophy in the dorsal part of the posterior cingulate cortex. Subtly different from the findings of previous studies, we identified the bilateral CTh of the dorsal part of the posterior cingulate gyrus as an important geometric feature to distinguish AD-related populations. Currently, there is a lack of research on the relationship between the insula circular sulcus and cognitive impairment. This study is the first to find that the GI of the left inferior segment of the insula circular sulcus is an important imaging feature to distinguish NC from EMCI.

Sauer et al. (2006) proposed that the number of STS neurons decreases by 50% in AD and that functional changes in the STS can be detected at the early stage of neuronal loss, prior to visible atrophy. Consistent with previous studies, we found that the CTh and SD of the left STS were important imaging features in the NC vs. EMCI and LMCI vs. (NC + EMCI) classification tasks, respectively.

Neuropsychological assessments provide essential information regarding the risk of cognitive impairment and remain the first line of choice for neurologists, whereas imaging features offer insight into cortical degeneration in AD. Uysal and Ozturk (2020) demonstrated that the efficient use of the brain with increasing age promotes the formation of new neuronal pathways and increases brain plasticity, resulting in elderly individuals with cortical atrophy but without cognitive impairment; this renders the performance of multi-class AD classification using structural MRI challenging. Although the subtlety of brain changes presents challenges for imaging-based classification, the combined use of clinical and imaging features is promising. Our study demonstrates that the combination of clinical and imaging features performs better than single features, suggesting that these features are both indispensable and complementary, thus leading to good diagnostic performance for AD.

### Hierarchical classification

Although researchers in the field of cognitive science have predominantly focused on relevant anatomical regions, high diagnostic accuracy remains essential for clinical purposes (Klöppel et al., 2012). Machine learning has gained recent interest for providing a second opinion for various neurodegenerative diseases, particularly for AD, which encompasses the majority of clinical neuroimaging research. To date, few studies have focused on cortical morphometry for classification tasks, let alone the multi-class of AD. Compared to the only two existing AD classification studies on cortical morphology, our results have higher accuracy, as shown in Table 4. Park et al. (2012) adopted CTh and SD as features for the implementation of simple multiple binary classifications. Liu et al. (2013) used the CTh of selected brain regions to differentiate NC from AD, and obtained an accuracy of 0.85. Bron et al. (2015) created an optimal algorithm with an accuracy of 0.63 using a combination of features, namely, volume, CTh, shape, and intensity on a multi-center dataset. Ma et al. (2016) used CTh as the classification feature for three-way classification and achieved a 0.65 accuracy, while Ma et al. (2020) utilized surface-based morphological measurements such as FD, SD, and CTh to distinguish NC from MCI, which did not improve classification accuracy in AD-related populations. The hierarchical multi-class framework established in our study shows good prospects for application in the auxiliary diagnosis of AD.

**TABLE 4:** Classification performance of different studies based on cortical morphological measurements.

References	Data set	Participants	Algorithm	Features	Overall accuracy
Park et al. (2012)	OASIS	25 AD/25 MCI/50 NC	SVM	CTh + SD	0.77 (AD/NC) 0.69 (MCI/NC) 0.63 (AD/MCI)
Liu et al. (2013)	ADNI	83 AD/137 NC	Elastic net + locally linear embedding	CTh	0.85 (AD/NC)
Bron et al. (2015)	Multi-center dataset	103 AD/122 MCI/129 NC	Sørensen-equal	Volume, thickness, shape, and intensity	0.63 (AD/MCI/NC)
Ma et al. (2016)	ADNI	15 AD/23 MCI/26 NC	SVM	CTh	0.65 (AD/MCI/NC)
Ma et al. (2020)	ADNI	30 MCI/16 NC	RF	CTh + FD + GI + SD	0.80
	Xuanwu	27 MCI/32 NC			0.80
Current work	ADNI	75 AD/83 LMCI/110 EMCI/417 NC	AdaBoost	CTh + FD + GI + SD + clinical features	0.877

AD, Alzheimer’s disease; MCI, mild cognitive impairment; EMCI, early mild cognitive impairment; LMCI, late mild cognitive impairment; NC, normal cognition; OASIS, the open access series of imaging studies; ADNI, Alzheimer’s Disease Neuroimaging Initiative; IXI, Information eXtraction from Images; SVM, support vector machine; CTh, cortical thickness; FD, fractal dimension; GI, gyrification index; SD, sulcus depth.

The current study has several limitations. First, the tgLASSO method we adopted for the model development required each participant to have corresponding structural MRI scans and MMSE scores at four different time points, which limited the size of our sample, owing to the concurrent need for both parameters. Second, due to their invasiveness, high cost, and poor availability, PET scans were not included in this study. Third, sample characteristics of the ADNI database resulted in differences between participants in the model development and the time validation set, the latter being younger and having more years of education. In future studies, we intend to improve our classification framework by expanding the sample size and including multimodal imaging data to enhance reliability, stability, and applicability for more comprehensive analyses.

This study developed an effective hierarchical multi-class framework with high accuracy, underscoring the utility of combining cognitive variables with imaging features and the reliability of surface-based morphometry. In conclusion, combining neuroimaging and clinical information with ML may facilitate more accurate early diagnosis of AD in clinical practice, reduce the unnecessary deployment of therapeutics, and streamline the workflow of clinicians, especially for cases requiring frequent monitoring or complex decision-making.

## Data availability statement

The datasets presented in this study can be found in online repositories. The names of the repository/repositories and accession number(s) can be found below: http://adni.loni.usc.edu.

## Ethics statement

The studies involving human participants were reviewed and approved by the Alzheimer’s Disease Neuroimaging Initiative. The patients/participants provided their written informed consent to participate in this study. Written informed consent was obtained from the individual(s) for the publication of any potentially identifiable images or data included in this article.

## The Alzheimer’s disease neuroimaging initiative (ADNI)

Data used in the preparation of this article were obtained from the Alzheimer’s Disease Neuroimaging Initiative (ADNI) database (adni.loni.usc.edu). As such, the investigators within the ADNI contributed to the design and implementation of ADNI and/or provided data but did not participate in the analysis or writing of this report. A complete listing of ADNI investigators can be found at: http://adni.loni.usc.edu/wp-content/uploads/how_to_apply/ADNI_Acknowledgement_List.pdf.

## Author contributions

YQ, XG, and HY contributed to the conception and design of the study. YQ, YT, HH, and JC organized the database. YQ, ZF, LL, and YL performed the statistical analysis. YQ wrote the manuscript. All authors contributed to manuscript revision, read, and approved the submitted version.

## Footnotes

1
adni.loni.usc.edu

2
http://www.neuro.uni-jena.de/cat/

3
http://www.fil.ion.ucl.ac.uk/spm/software/spm12/
