# Supplementary material for: Hierarchical multi-class Alzheimer’s disease diagnostic framework using imaging and clinical features
Source: Front Aging Neurosci. 2022 Aug 10;14:935055. doi: 10.3389/fnagi.2022.935055 (PMC9399682; doi:10.3389/fnagi.2022.935055)
Supplement: Supplementary file 1 [file Data_Sheet_1.docx]

Table 1

Hierarchical multi-class results of clinical features (including MMSE and CDR), imaging features and combined features in “NC-EMCI-LMCI-AD” scenario using AdaBoost algorithm (clinical_r refers to clinical features removing MMSE and CDR)

| Datase | Features | Classifiers | SEN | SPE | Accuracy | B-accuracy | F1 | AUC |
| --- | --- | --- | --- | --- | --- | --- | --- | --- |
| Model development | clinical+imaging | NC vs. (EMCI+LMCI+AD) | 0.956 | 0.991 | 0.973 | 0.974 | 0.972 | 0.993 |
|  |  | EMCI vs. (LMCI+AD) | 0.866 | 0.813 | 0.836 | 0.840 | 0.840 | 0.911 |
|  |  | LMCI vs. AD | 0.955 | 0.940 | 0.946 | 0.948 | 0.945 | 0.983 |
|  | clinical_r+imaging | NC vs. (EMCI+LMCI+AD) | 0.867 | 0.764 | 0.816 | 0.816 | 0.825 | 0.874 |
|  |  | EMCI vs. (LMCI+AD) | 0.849 | 0.792 | 0.817 | 0.821 | 0.821 | 0.896 |
|  |  | LMCI vs. AD | 0.828 | 0.868 | 0.843 | 0.848 | 0.836 | 0.923 |
|  | clinical | NC vs. (EMCI+LMCI+AD) | 0.972 | 0.986 | 0.979 | 0.979 | 0.978 | 0.994 |
|  |  | EMCI vs. (LMCI+AD) | 0.862 | 0.602 | 0.731 | 0.732 | 0.759 | 0.798 |
|  |  | LMCI vs. AD | 0.945 | 0.929 | 0.935 | 0.937 | 0.937 | 0.983 |
|  | clinical_r | NC vs. (EMCI+LMCI+AD) | 0.905 | 0.736 | 0.822 | 0.821 | 0.835 | 0.885 |
|  |  | EMCI vs. (LMCI+AD) | 0.813 | 0.606 | 0.709 | 0.710 | 0.733 | 0.774 |
|  |  | LMCI vs. AD | 0.849 | 0.856 | 0.847 | 0.853 | 0.845 | 0.908 |
|  | imaging | NC vs. (EMCI+LMCI+AD) | 0.693 | 0.656 | 0.673 | 0.675 | 0.677 | 0.759 |
|  |  | EMCI vs. (LMCI+AD) | 0.821 | 0.801 | 0.807 | 0.811 | 0.807 | 0.872 |
|  |  | LMCI vs. AD | 0.728 | 0.706 | 0.718 | 0.717 | 0.717 | 0.779 |
| Temporal validation | clinical+imaging | NC vs. (EMCI+LMCI+AD) | 0.935 | 0.922 | 0.930 | 0.928 | 0.942 | 0.935 |
|  |  | EMCI vs. (LMCI+AD) | 0.809 | 0.658 | 0.720 | 0.734 | 0.704 | 0.809 |
|  |  | LMCI vs. AD | 0.831 | 0.907 | 0.867 | 0.869 | 0.868 | 0.831 |
|  | clinical_r+imaging | NC vs. (EMCI+LMCI+AD) | 0.892 | 0.675 | 0.807 | 0.784 | 0.849 | 0.892 |
|  |  | EMCI vs. (LMCI+AD) | 0.764 | 0.646 | 0.694 | 0.705 | 0.672 | 0.764 |
|  |  | LMCI vs. AD | 0.783 | 0.813 | 0.798 | 0.798 | 0.802 | 0.783 |
|  | clinical | NC vs. (EMCI+LMCI+AD) | 0.871 | 0.896 | 0.880 | 0.883 | 0.899 | 0.871 |
|  |  | EMCI vs. (LMCI+AD) | 0.673 | 0.652 | 0.660 | 0.662 | 0.619 | 0.673 |
|  |  | LMCI vs. AD | 0.783 | 0.920 | 0.848 | 0.852 | 0.844 | 0.783 |
|  | clinical_r | NC vs. (EMCI+LMCI+AD) | 0.765 | 0.724 | 0.749 | 0.744 | 0.788 | 0.765 |
|  |  | EMCI vs. (LMCI+AD) | 0.591 | 0.677 | 0.642 | 0.634 | 0.575 | 0.591 |
|  |  | LMCI vs. AD | 0.771 | 0.813 | 0.791 | 0.792 | 0.795 | 0.771 |
|  | imaging | NC vs. (EMCI+LMCI+AD) | 0.648 | 0.638 | 0.644 | 0.643 | 0.689 | 0.648 |
|  |  | EMCI vs. (LMCI+AD) | 0.573 | 0.595 | 0.586 | 0.584 | 0.532 | 0.573 |
|  |  | LMCI vs. AD | 0.723 | 0.653 | 0.690 | 0.688 | 0.710 | 0.723 |

AD: Alzheimer's disease, EMCI: early mild cognitive impairment, LMCI: late mild cognitive impairment, NC: normal cognition, SEN: sensitivity, SPE: specificity, B-accuracy: balanced accuracy, AUC: area under the curve.

Table 2

Hierarchical multi-class results of clinical features, imaging features and combined features in “NC-AD-EMCI-LMCI” scenario using AdaBoost algorithm (clinical_r refers to clinical features removing MMSE and CDR)

| Datase | Features | Classifiers | SEN | SPE | Accuracy | B-accuracy | F1 | AUC |
| --- | --- | --- | --- | --- | --- | --- | --- | --- |
| Model development | clinical+imaging | NC vs. (EMCI+LMCI+AD) | 0.956 | 0.991 | 0.973 | 0.974 | 0.972 | 0.993 |
|  |  | AD vs. (EMCI+LMCI) | 0.966 | 0.952 | 0.958 | 0.959 | 0.960 | 0.988 |
|  |  | EMCI vs. LMCI | 0.689 | 0.691 | 0.693 | 0.690 | 0.683 | 0.750 |
|  | clinical_r+imaging | NC vs. (EMCI+LMCI+AD) | 0.867 | 0.764 | 0.816 | 0.816 | 0.825 | 0.874 |
|  |  | AD vs. (EMCI+LMCI) | 0.890 | 0.877 | 0.883 | 0.884 | 0.882 | 0.948 |
|  |  | EMCI vs. LMCI | 0.702 | 0.661 | 0.684 | 0.682 | 0.683 | 0.731 |
|  | clinical | NC vs. (EMCI+LMCI+AD) | 0.972 | 0.986 | 0.979 | 0.979 | 0.978 | 0.994 |
|  |  | AD vs. (EMCI+LMCI) | 0.976 | 0.940 | 0.957 | 0.958 | 0.958 | 0.985 |
|  |  | EMCI vs. LMCI | 0.617 | 0.557 | 0.582 | 0.587 | 0.592 | 0.633 |
|  | clinical_r | NC vs. (EMCI+LMCI+AD) | 0.905 | 0.736 | 0.822 | 0.821 | 0.835 | 0.885 |
|  |  | AD vs. (EMCI+LMCI) | 0.885 | 0.861 | 0.869 | 0.873 | 0.870 | 0.932 |
|  |  | EMCI vs. LMCI | 0.600 | 0.545 | 0.567 | 0.573 | 0.573 | 0.583 |
|  | imaging | NC vs. (EMCI+LMCI+AD) | 0.693 | 0.656 | 0.673 | 0.675 | 0.677 | 0.759 |
|  |  | AD vs. (EMCI+LMCI) | 0.851 | 0.785 | 0.819 | 0.818 | 0.822 | 0.891 |
|  |  | EMCI vs. LMCI | 0.717 | 0.669 | 0.695 | 0.693 | 0.696 | 0.744 |
| Temporal validation | clinical+imaging | NC vs. (EMCI+LMCI+AD) | 0.978 | 0.731 | 0.882 | 0.855 | 0.910 | 0.855 |
|  |  | AD vs. (EMCI+LMCI) | 0.947 | 0.886 | 0.903 | 0.917 | 0.845 | 0.916 |
|  |  | EMCI vs. LMCI | 0.682 | 0.494 | 0.601 | 0.588 | 0.661 | 0.588 |
|  | clinical_r+imaging | NC vs. (EMCI+LMCI+AD) | 0.983 | 0.444 | 0.772 | 0.714 | 0.840 | 0.714 |
|  |  | AD vs. (EMCI+LMCI) | 0.813 | 0.876 | 0.858 | 0.845 | 0.763 | 0.845 |
|  |  | EMCI vs. LMCI | 0.718 | 0.434 | 0.596 | 0.576 | 0.669 | 0.576 |
|  | clinical | NC vs. (EMCI+LMCI+AD) | 0.989 | 0.746 | 0.882 | 0.868 | 0.909 | 0.858 |
|  |  | AD vs. (EMCI+LMCI) | 0.907 | 0.891 | 0.896 | 0.899 | 0.829 | 0.899 |
|  |  | EMCI vs. LMCI | 0.582 | 0.446 | 0.523 | 0.514 | 0.582 | 0.514 |
|  | clinical_r | NC vs. (EMCI+LMCI+AD) | 0.799 | 0.653 | 0.742 | 0.726 | 0.790 | 0.726 |
|  |  | AD vs. (EMCI+LMCI) | 0.840 | 0.834 | 0.836 | 0.837 | 0.741 | 0.837 |
|  |  | EMCI vs. LMCI | 0.600 | 0.434 | 0.529 | 0.517 | 0.592 | 0.517 |
|  | imaging | NC vs. (EMCI+LMCI+AD) | 0.729 | 0.593 | 0.676 | 0.661 | 0.733 | 0.661 |
|  |  | AD vs. (EMCI+LMCI) | 0.720 | 0.788 | 0.769 | 0.754 | 0.635 | 0.754 |
|  |  | EMCI vs. LMCI | 0.591 | 0.446 | 0.529 | 0.519 | 0.588 | 0.518 |

AD: Alzheimer's disease, EMCI: early mild cognitive impairment, LMCI: late mild cognitive impairment, NC: normal cognition, SEN: sensitivity, SPE: specificity, B-accuracy: balanced accuracy, AUC: area under the curve.

Table 3

Hierarchical multi-class results of clinical features, imaging features and combined features in “AD-LMCI-EMCI-NC” scenario using AdaBoost algorithm (clinical_r refers to clinical features removing MMSE and CDR)

| Datase | Features | Classifiers | SEN | SPE | Accuracy | B-accuracy | F1 | AUC |
| --- | --- | --- | --- | --- | --- | --- | --- | --- |
| Model development | clinical+imaging | AD vs. (NC+EMCI+LMCI) | 0.996 | 0.972 | 0.984 | 0.984 | 0.983 | 0.994 |
|  |  | LMCI vs. (NC+EMCI) | 0.895 | 0.765 | 0.829 | 0.830 | 0.839 | 0.894 |
|  |  | NC vs. EMCI | 0.934 | 1.000 | 0.967 | 0.967 | 0.965 | 0.983 |
|  | clinical_r+imaging | AD vs. (NC+EMCI+LMCI) | 0.942 | 0.916 | 0.930 | 0.929 | 0.930 | 0.945 |
|  |  | LMCI vs. (NC+EMCI) | 0.757 | 0.742 | 0.749 | 0.750 | 0.751 | 0.806 |
|  |  | NC vs. EMCI | 0.800 | 0.751 | 0.774 | 0.776 | 0.780 | 0.857 |
|  | clinical | AD vs. (NC+EMCI+LMCI) | 0.979 | 0.972 | 0.976 | 0.976 | 0.975 | 0.992 |
|  |  | LMCI vs. (NC+EMCI) | 0.903 | 0.662 | 0.782 | 0.783 | 0.805 | 0.829 |
|  |  | NC vs. EMCI | 0.937 | 1.000 | 0.969 | 0.969 | 0.967 | 0.987 |
|  | clinical_r | AD vs. (NC+EMCI+LMCI) | 0.947 | 0.884 | 0.915 | 0.916 | 0.916 | 0.956 |
|  |  | LMCI vs. (NC+EMCI) | 0.620 | 0.723 | 0.671 | 0.672 | 0.650 | 0.718 |
|  |  | NC vs. EMCI | 0.828 | 0.707 | 0.768 | 0.768 | 0.781 | 0.835 |
|  | imaging | AD vs. (NC+EMCI+LMCI) | 0.890 | 0.863 | 0.877 | 0.877 | 0.877 | 0.937 |
|  |  | LMCI vs. (NC+EMCI) | 0.707 | 0.685 | 0.697 | 0.696 | 0.699 | 0.740 |
|  |  | NC vs. EMCI | 0.580 | 0.618 | 0.600 | 0.599 | 0.590 | 0.646 |
| Temporal validation | clinical+imaging | AD vs. (NC+EMCI+LMCI) | 0.933 | 0.956 | 0.953 | 0.945 | 0.814 | 0.945 |
|  |  | LMCI vs. (NC+EMCI) | 0.711 | 0.820 | 0.805 | 0.766 | 0.498 | 0.765 |
|  |  | NC vs. EMCI | 0.897 | 0.891 | 0.896 | 0.894 | 0.932 | 0.894 |
|  | clinical_r+imaging | AD vs. (NC+EMCI+LMCI) | 1.000 | 0.020 | 0.127 | 0.510 | 0.201 | 0.510 |
|  |  | LMCI vs. (NC+EMCI) | 0.193 | 0.863 | 0.772 | 0.528 | 0.187 | 0.528 |
|  |  | NC vs. EMCI | 1.000 | 0.000 | 0.791 | 0.500 | 0.883 | 0.500 |
|  | clinical | AD vs. (NC+EMCI+LMCI) | 0.920 | 0.956 | 0.952 | 0.938 | 0.807 | 0.938 |
|  |  | LMCI vs. (NC+EMCI) | 0.036 | 0.994 | 0.864 | 0.515 | 0.067 | 0.515 |
|  |  | NC vs. EMCI | 0.894 | 0.890 | 0.894 | 0.892 | 0.930 | 0.893 |
|  | clinical_r | AD vs. (NC+EMCI+LMCI) | 0.440 | 0.990 | 0.930 | 0.715 | 0.579 | 0.715 |
|  |  | LMCI vs. (NC+EMCI) | 0.590 | 0.795 | 0.767 | 0.693 | 0.408 | 0.693 |
|  |  | NC vs. EMCI | 0.998 | 0.055 | 0.801 | 0.527 | 0.888 | 0.526 |
|  | imaging | AD vs. (NC+EMCI+LMCI) | 0.867 | 0.912 | 0.907 | 0.890 | 0.670 | 0.889 |
|  |  | LMCI vs. (NC+EMCI) | 0.663 | 0.791 | 0.774 | 0.727 | 0.727 | 0.444 |
|  |  | NC vs. EMCI | 0.635 | 0.627 | 0.634 | 0.631 | 0.733 | 0.631 |

AD: Alzheimer's disease, EMCI: early mild cognitive impairment, LMCI: late mild cognitive impairment, NC: normal cognition, SEN: sensitivity, SPE: specificity, B-accuracy: balanced accuracy, AUC: area under the curve.

Table 4

Hierarchical multi-class results of clinical features, imaging features and combined features in “AD-NC-EMCI-LMCI” scenario using AdaBoost algorithm (clinical_r refers to clinical features removing MMSE and CDR)

| Dataset | Features | Classifiers | SEN | SPE | Accuracy | B-accuracy | F1 | AUC |
| --- | --- | --- | --- | --- | --- | --- | --- | --- |
| Model development | clinical+imaging | AD vs. (NC+EMCI+LMCI) | 0.942 | 0.916 | 0.930 | 0.929 | 0.930 | 0.975 |
|  |  | NC vs. (EMCI+LMCI) | 0.941 | 0.998 | 0.969 | 0.970 | 0.968 | 0.987 |
|  |  | EMCI vs. LMCI | 0.689 | 0.691 | 0.693 | 0.690 | 0.683 | 0.750 |
|  | clinical_r+imaging | AD vs. (NC+EMCI+LMCI) | 0.942 | 0.916 | 0.930 | 0.929 | 0.930 | 0.975 |
|  |  | NC vs. (EMCI+LMCI) | 0.830 | 0.686 | 0.759 | 0.758 | 0.775 | 0.817 |
|  |  | EMCI vs. LMCI | 0.702 | 0.661 | 0.684 | 0.682 | 0.683 | 0.731 |
|  | clinical | AD vs. (NC+EMCI+LMCI) | 0.979 | 0.972 | 0.976 | 0.976 | 0.975 | 0.992 |
|  |  | NC vs. (EMCI+LMCI) | 0.941 | 0.990 | 0.965 | 0.966 | 0.964 | 0.988 |
|  |  | EMCI vs. LMCI | 0.617 | 0.557 | 0.582 | 0.587 | 0.592 | 0.633 |
|  | clinical_r | AD vs. (NC+EMCI+LMCI) | 0.947 | 0.884 | 0.888 | 0.916 | 0.916 | 0.956 |
|  |  | NC vs. (EMCI+LMCI) | 0.842 | 0.724 | 0.783 | 0.783 | 0.793 | 0.836 |
|  |  | EMCI vs. LMCI | 0.599 | 0.545 | 0.567 | 0.572 | 0.573 | 0.583 |
|  | imaging | AD vs. (NC+EMCI+LMCI) | 0.890 | 0.863 | 0.877 | 0.877 | 0.877 | 0.937 |
|  |  | NC vs. (EMCI+LMCI) | 0.577 | 0.585 | 0.580 | 0.581 | 0.577 | 0.610 |
|  |  | EMCI vs. LMCI | 0.717 | 0.669 | 0.695 | 0.693 | 0.696 | 0.744 |
| Temporal validation | clinical+imaging | AD vs. (NC+EMCI+LMCI) | 0.947 | 0.957 | 0.956 | 0.952 | 0.826 | 0.952 |
|  |  | NC vs. (EMCI+LMCI) | 0.962 | 0.772 | 0.902 | 0.867 | 0.930 | 0.867 |
|  |  | EMCI vs. LMCI | 0.682 | 0.494 | 0.601 | 0.588 | 0.661 | 0.588 |
|  | clinical_r+imaging | AD vs. (NC+EMCI+LMCI) | 0.840 | 0.946 | 0.934 | 0.893 | 0.934 | 0.893 |
|  |  | NC vs. (EMCI+LMCI) | 0.909 | 0.497 | 0.779 | 0.703 | 0.849 | 0.703 |
|  |  | EMCI vs. LMCI | 0.718 | 0.434 | 0.596 | 0.576 | 0.669 | 0.576 |
|  | clinical | AD vs. (NC+EMCI+LMCI) | 0.840 | 0.926 | 0.917 | 0.883 | 0.689 | 0.883 |
|  |  | NC vs. (EMCI+LMCI) | 0.952 | 0.845 | 0.918 | 0.899 | 0.941 | 0.898 |
|  |  | EMCI vs. LMCI | 0.582 | 0.446 | 0.523 | 0.514 | 0.582 | 0.514 |
|  | clinical_r | AD vs. (NC+EMCI+LMCI) | 0.840 | 0.926 | 0.917 | 0.883 | 0.689 | 0.883 |
|  |  | NC vs. (EMCI+LMCI) | 0.731 | 0.653 | 0.707 | 0.692 | 0.773 | 0.692 |
|  |  | EMCI vs. LMCI | 0.600 | 0.434 | 0.529 | 0.517 | 0.592 | 0..517 |
|  | imaging | AD vs. (NC+EMCI+LMCI) | 0.693 | 0.871 | 0.851 | 0.782 | 0.505 | 0.782 |
|  |  | NC vs. (EMCI+LMCI) | 0.669 | 0.513 | 0.620 | 0.591 | 0.706 | 0.591 |
|  |  | EMCI vs. LMCI | 0.591 | 0.446 | 0.529 | 0.519 | 0.588 | 0.518 |

AD: Alzheimer's disease, EMCI: early mild cognitive impairment, LMCI: late mild cognitive impairment, NC: normal cognition, SEN: sensitivity, SPE: specificity, B-accuracy: balanced accuracy, AUC: area under the curve.

Table 5

Tuning procedures of several machine learning methods

| Algorithms | Tuning procedures |
| --- | --- |
| AdaBoost | n_estimators: [0,50,500], learning rate: [0,1,10] |
| Bagging | minimum out-of-bag error |
| K-nearest neighbor | kernel: rectangular, kmax: 50, distance: 1 |
| Logistic Regression | family: binomial |
| Naive Bayes | Laplace: 0 |
| Random Fores | minimum out-of-bag error |
| Support Vector Machine | kernel: linear, gamma: 10^(-2:1), cost: [0.001, 0.01, 0.1, 1, 5, 10] |

Table 6

Detailed final values of hyperparameters for several machine learning methods in “NC-EMCI-LMCI-AD” scenario (clinical_r refers to clinical features removing MMSE and CDR)

| Features | Classifiers | AdaBoost | | Bagging | | KNN | RF | | | SVM | |
| --- | --- | --- | --- | --- | --- | --- | --- | --- | --- | --- | --- |
|  |  | n_estimators | learning_rate | mtry | min.err | best k | mtry | min.err | nodesize | gamma | cost |
| clinical  +imaging | NC vs. (EMCI+LMCI+AD) | 250 | 0.111 | 7 | 167 | 2 | 7 | 346 | 5 | 0.01 | 5 |
|  | EMCI vs. (LMCI+AD) | 450 | 1 | 7 | 175 | 2 | 7 | 449 | 5 | 0.01 | 0.01 |
|  | LMCI vs. AD | 100 | 0.111 | 7 | 319 | 20 | 7 | 364 | 5 | 0.01 | 0.01 |
| clinical_r  +imaging | NC vs. (EMCI+LMCI+AD) | 100 | 0.667 | 7 | 152 | 2 | 7 | 390 | 5 | 0.01 | 10 |
|  | EMCI vs. (LMCI+AD) | 200 | 0.444 | 7 | 426 | 4 | 7 | 148 | 5 | 0.01 | 0.1 |
|  | LMCI vs. AD | 100 | 0.111 | 7 | 363 | 16 | 7 | 433 | 5 | 0.01 | 0.01 |
| clinical | NC vs. (EMCI+LMCI+AD) | 400 | 0.333 | 2 | 29 | 1 | 2 | 1 | 5 | 0.01 | 10 |
|  | EMCI vs. (LMCI+AD) | 50 | 0.111 | 2 | 109 | 36 | 2 | 373 | 5 | 0.01 | 1 |
|  | LMCI vs. AD | 150 | 0.111 | 2 | 72 | 22 | 2 | 57 | 5 | 0.01 | 0.1 |
| clinical_r | NC vs. (EMCI+LMCI+AD) | 50 | 0.111 | 2 | 269 | 10 | 2 | 157 | 5 | 0.01 | 5 |
|  | EMCI vs. (LMCI+AD) | 50 | 0.444 | 2 | 473 | 1 | 2 | 61 | 5 | 0.01 | 5 |
|  | LMCI vs. AD | 300 | 0.111 | 2 | 42 | 14 | 2 | 24 | 5 | 0.01 | 0.1 |
| imaging | NC vs. (EMCI+LMCI+AD) | 50 | 1 | 6 | 115 | 1 | 6 | 99 | 5 | 0.01 | 0.001 |
|  | EMCI vs. (LMCI+AD) | 400 | 0.444 | 6 | 217 | 14 | 6 | 492 | 5 | 0.01 | 0.01 |
|  | LMCI vs. AD | 150 | 0.222 | 6 | 237 | 36 | 6 | 481 | 5 | 0.01 | 0.01 |

AD: Alzheimer's disease, EMCI: early mild cognitive impairment, LMCI: late mild cognitive impairment, NC: normal cognition, KNN: K-nearest neighbor, RF: Random Forest, SVM: Support Vector Machine.

Table 7

Detailed final values of hyperparameters for several machine learning methods in “NC-AD-EMCI-LMCI” scenario (clinical_r refers to clinical features removing MMSE and CDR)

| Features | Classifiers | AdaBoost | | Bagging | | KNN | RF | | | SVM | |
| --- | --- | --- | --- | --- | --- | --- | --- | --- | --- | --- | --- |
|  |  | n_estimators | learning_rate | mtry | min.err | best k | mtry | min.err | nodesize | gamma | cost |
| clinical+imaging | NC vs. (EMCI+LMCI+AD) | 250 | 0.111 | 7 | 167 | 2 | 7 | 346 | 5 | 0.01 | 5 |
|  | AD vs. (EMCI+LMCI) | 450 | 1 | 7 | 174 | 1 | 7 | 92 | 5 | 0.01 | 0.01 |
|  | EMCI vs. LMCI | 100 | 0.333 | 7 | 327 | 12 | 7 | 293 | 5 | 0.01 | 0.01 |
| clinical_r+imaging | NC vs. (EMCI+LMCI+AD) | 100 | 0.667 | 7 | 152 | 2 | 7 | 390 | 5 | 0.01 | 10 |
|  | AD vs. (EMCI+LMCI) | 450 | 1 | 7 | 322 | 3 | 7 | 304 | 5 | 0.01 | 0.01 |
|  | EMCI vs. LMCI | 450 | 0.333 | 7 | 455 | 10 | 7 | 348 | 5 | 0.01 | 0.01 |
| clinical | NC vs. (EMCI+LMCI+AD) | 400 | 0.333 | 2 | 29 | 1 | 2 | 1 | 5 | 0.01 | 10 |
|  | AD vs. (EMCI+LMCI) | 350 | 1 | 2 | 21 | 5 | 2 | 51 | 5 | 0.01 | 0.01 |
|  | EMCI vs. LMCI | 100 | 0.556 | 2 | 328 | 3 | 2 | 87 | 5 | 0.01 | 1 |
| clinical_r | NC vs. (EMCI+LMCI+AD) | 50 | 0.111 | 2 | 269 | 10 | 2 | 157 | 5 | 0.01 | 5 |
|  | AD vs. (EMCI+LMCI) | 450 | 0.111 | 2 | 56 | 7 | 2 | 62 | 5 | 0.01 | 1 |
|  | EMCI vs. LMCI | 50 | 0.556 | 2 | 90 | 5 | 2 | 73 | 5 | 0.01 | 1 |
| imaging | NC vs. (EMCI+LMCI+AD) | 50 | 1 | 6 | 115 | 1 | 6 | 99 | 5 | 0.01 | 0.001 |
|  | AD vs. (EMCI+LMCI) | 450 | 0.222 | 6 | 130 | 13 | 6 | 330 | 5 | 0.01 | 0.01 |
|  | EMCI vs. LMCI | 450 | 0.222 | 6 | 190 | 14 | 6 | 415 | 5 | 0.01 | 0.01 |

AD: Alzheimer's disease, EMCI: early mild cognitive impairment, LMCI: late mild cognitive impairment, NC: normal cognition, KNN: K-nearest neighbor, RF: Random Forest, SVM: Support Vector Machine.

Table 8

Detailed final values of hyperparameters for several machine learning methods in “AD-LMCI-NC-EMCI” scenario (clinical_r refers to clinical features removing MMSE and CDR)

| Features | Classifiers | AdaBoost | | Bagging | | KNN | RF | | | SVM | |
| --- | --- | --- | --- | --- | --- | --- | --- | --- | --- | --- | --- |
|  |  | n_estimators | learning_rate | mtry | min.err | best k | mtry | min.err | nodesize | gamma | cost |
| clinical  +imaging | AD vs. (NC+EMCI+LMCI) | 450 | 0.556 | 7 | 230 | 2 | 7 | 194 | 5 | 0.01 | 0.01 |
|  | LMCI vs. (NC+EMCI) | 50 | 0.333 | 7 | 337 | 3 | 7 | 389 | 5 | 0.01 | 0.01 |
|  | NC vs. EMCI | 50 | 0.111 | 7 | 272 | 16 | 7 | 469 | 5 | 0.01 | 0.1 |
| clinical_r  +imaging | AD vs. (NC+EMCI+LMCI) | 450 | 0.778 | 7 | 262 | 2 | 7 | 411 | 5 | 0.01 | 0.1 |
|  | LMCI vs. (NC+EMCI) | 450 | 0.111 | 7 | 435 | 1 | 7 | 195 | 5 | 0.01 | 0.01 |
|  | NC vs. EMCI | 450 | 0.111 | 7 | 260 | 1 | 7 | 389 | 5 | 0.01 | 10 |
| clinical | AD vs. (NC+EMCI+LMCI) | 400 | 0.222 | 2 | 67 | 5 | 2 | 127 | 5 | 0.01 | 0.01 |
|  | LMCI vs. (NC+EMCI) | 50 | 0.778 | 2 | 288 | 3 | 2 | 31 | 5 | 0.01 | 5 |
|  | NC vs. EMCI | 50 | 0.111 | 2 | 1 | 4 | 2 | 137 | 5 | 0.01 | 1 |
| clinical_r | AD vs. (NC+EMCI+LMCI) | 100 | 0.778 | 2 | 477 | 3 | 2 | 214 | 5 | 0.01 | 1 |
|  | LMCI vs. (NC+EMCI) | 50 | 0.111 | 2 | 210 | 1 | 2 | 285 | 5 | 0.01 | 1 |
|  | NC vs. EMCI | 50 | 0.778 | 2 | 54 | 18 | 2 | 104 | 5 | 0.01 | 10 |
| imaging | AD vs. (NC+EMCI+LMCI) | 400 | 0.556 | 6 | 387 | 1 | 6 | 493 | 5 | 0.01 | 0.1 |
|  | LMCI vs. (NC+EMCI) | 150 | 0.556 | 6 | 383 | 17 | 6 | 98 | 5 | 0.01 | 0.001 |
|  | NC vs. EMCI | 200 | 1 | 6 | 117 | 1 | 6 | 267 | 5 | 0.01 | 0.1 |

AD: Alzheimer's disease, EMCI: early mild cognitive impairment, LMCI: late mild cognitive impairment, NC: normal cognition, KNN: K-nearest neighbor, RF: Random Forest, SVM: Support Vector Machine.

Table 9

Detailed final values of hyperparameters for several machine learning methods in “AD-NC-EMCI-LMCI” scenario (clinical_r refers to clinical features removing MMSE and CDR)

| Features | Classifiers | AdaBoost | | Bagging | | KNN | RF | | | SVM | |
| --- | --- | --- | --- | --- | --- | --- | --- | --- | --- | --- | --- |
|  |  | n_estimators | learning_rate | mtry | min.err | best k | mtry | min.err | nodesize | gamma | cost |
| clinical  +imaging | AD vs. (NC+EMCI+LMCI) | 450 | 0.778 | 7 | 262 | 2 | 7 | 411 | 5 | 0.01 | 0.1 |
|  | NC vs. (EMCI+LMCI) | 150 | 0.111 | 7 | 98 | 22 | 7 | 404 | 5 | 0.01 | 0.1 |
|  | EMCI vs. LMCI | 100 | 0.333 | 7 | 327 | 12 | 7 | 293 | 5 | 0.01 | 0.01 |
| clinical_r  +imaging | AD vs. (NC+EMCI+LMCI) | 450 | 0.778 | 7 | 262 | 2 | 7 | 411 | 5 | 0.01 | 0.1 |
|  | NC vs. (EMCI+LMCI) | 150 | 0.111 | 7 | 93 | 2 | 7 | 493 | 5 | 0.01 | 10 |
|  | EMCI vs. LMCI | 450 | 0.333 | 7 | 455 | 10 | 7 | 348 | 5 | 0.01 | 0.01 |
| clinical | AD vs. (NC+EMCI+LMCI) | 400 | 0.222 | 2 | 67 | 5 | 2 | 127 | 5 | 0.01 | 0.01 |
|  | NC vs. (EMCI+LMCI) | 100 | 0.111 | 2 | 306 | 2 | 2 | 40 | 5 | 0.01 | 0.1 |
|  | EMCI vs. LMCI | 100 | 0.556 | 2 | 328 | 3 | 2 | 87 | 5 | 0.01 | 1 |
| clinical_r | AD vs. (NC+EMCI+LMCI) | 100 | 0.778 | 2 | 477 | 3 | 2 | 214 | 5 | 0.01 | 1 |
|  | NC vs. (EMCI+LMCI) | 200 | 0.889 | 2 | 158 | 26 | 2 | 212 | 5 | 0.01 | 5 |
|  | EMCI vs. LMCI | 50 | 0.556 | 2 | 90 | 5 | 2 | 73 | 5 | 0.01 | 1 |
| imaging | AD vs. (NC+EMCI+LMCI) | 400 | 0.556 | 6 | 387 | 1 | 6 | 493 | 5 | 0.01 | 0.1 |
|  | NC vs. (EMCI+LMCI) | 150 | 0.778 | 6 | 294 | 1 | 6 | 131 | 5 | 0.01 | 0.001 |
|  | EMCI vs. LMCI | 450 | 0.222 | 6 | 190 | 14 | 6 | 415 | 5 | 0.01 | 0.01 |

AD: Alzheimer's disease, EMCI: early mild cognitive impairment, LMCI: late mild cognitive impairment, NC: normal cognition, KNN: K-nearest neighbor, RF: Random Forest, SVM: Support Vector Machine.

Figure 1

Features importance scores of hierarchical binary classification tasks in AD-LMCI-EMCI-NC scenario


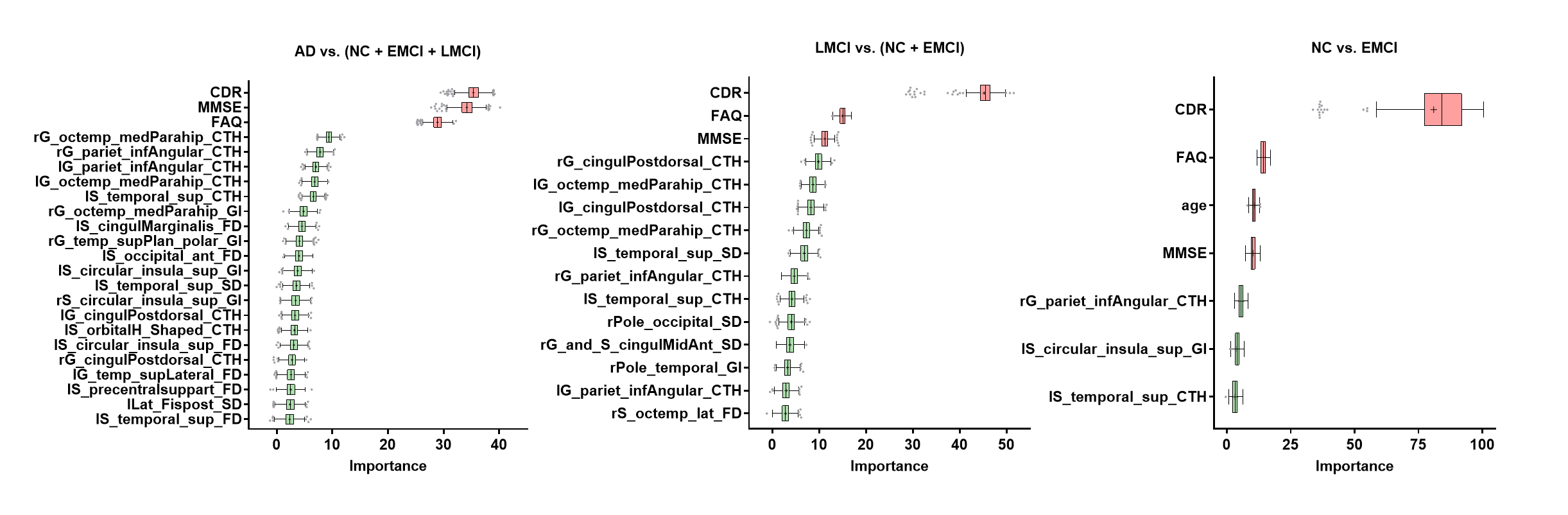
AD, Alzheimer's disease; MCI, mild cognitive impairment; EMCI, early mild cognitive impairment; LMCI, late mild cognitive impairment; NC, normal cognition; CTh, cortical thickness; FD, fractal dimension; GI, gyrification index; SD, sulcus depth; CDR, Clinical Dementia Rating; FAQ, Functional Activities Questionnaire; MMSE, mini-mental state exam.
